# Randomized clinical trial on the effect of intermittent vibrational force application during orthodontic treatment with aligners on RANKL and OPG concentrations in crevicular fluid

**DOI:** 10.1002/btm2.10491

**Published:** 2023-04-01

**Authors:** Alicia Pérez Idarraga, Fara Yeste Ojeda, Leire Virto Ruiz, Miguel Lacasa Litner, Alberto Cacho Casado, Conchita Martin

**Affiliations:** ^1^ Department of Orthodontics, Faculty of Odontology University Complutense Madrid Madrid Spain; ^2^ Department of Orthodontics Universidad San Pablo CEU Madrid Spain; ^3^ ETEP (Etiology and Therapy of Periodontal and Peri‐implant Diseases), Research Group Universidad Complutense Madrid Madrid Spain; ^4^ Department of Orthodontics CEPUME, Universidad de Alcalá de Henares, Madrid Madrid Spain; ^5^ BIOCRAN (Craniofacial Biology: Orthodontics and Dentofacial Orthopedics), Research Group University Complutense Madrid Madrid Spain

**Keywords:** accelerated tooth movement, aligners, biomarkers, gingival crevicular fluid, tooth movement techniques, vibration

## Abstract

Application of intermittent forces by vibration is proposed as an easy‐to‐use accelerator of dental movement. The purpose of this study was to determine the effect of intermittent vibrational force application during orthodontic aligner treatment on receptor activator of nuclear factor‐kappa B ligand (RANKL) and osteoprotegerin (OPG) concentrations in crevicular fluid as markers of bone remodeling. This three‐arm parallel randomized clinical trial included 45 candidates for malocclusion treatment with aligners, randomly assigned to: Group A (vibrational forces from onset of treatment); Group B (vibrational forces at 6 weeks after treatment onset); or Group C (no vibration). The frequency of aligner adjustment also differed among groups. At different time points, a paper tip was used to draw crevicular fluid samples from a moving lower incisor for RANKL and OPG analysis using ELISA kits. Mixed‐model ANOVA found no significant differences in RANKL (A: *p* = 0.31, B: *p* = 0.8, C: *p* = 0.49) or OPG (A: *p* = 0.24, B: *p* = 0.58, C: *p* = 0.59) over time in any group or as a function of the application/non‐application of vibration or the frequency of aligner adjustments. Application of this accelerator device did not significantly affect bone remodeling in patients undergoing orthodontic treatment with aligners. However, a nonsignificant improvement in biomarker concentrations was observed when aligners were changed every 7 days and vibration was also applied. Further research is warranted to establish protocols for the application of vibration and the timing of aligner adjustments.

## 1 BACKGROUND

The growing number of adults seeking orthodontic therapy has focused interest on the acceleration of tooth movement to reduce treatment times. Longer treatments carry a higher risk of gingivitis, decalcifications, caries, and possible root resorption and have a greater negative impact on the quality of life and facial esthetics of patients.^1^, ^2^ Surgical approaches to acceleration have been associated with elevated morbidity, and the application of intermittent forces by vibration offers an alternative easy‐to‐use option that is well accepted by patients and may have similar cellular effects.

Interactions of numerous inflammation mediators and growth factors have been related to cellular activation and responses after tooth movement onset. These biomediators and inflammatory markers are involved not only in activating osteoclasts and osteoblasts during bone remodeling but also in regulating the speed of tooth movement.^3^ The main mediators reported to regulate bone remodeling during orthodontic treatment are interleukin 1β, tumor necrosis factor, receptor activator of nuclear factor‐kappa B ligand (RANKL), receptor activator of nuclear factor‐kappa B (RANK), and osteoprotegerin (OPG).^4^, ^5^, ^6^ Bone remodeling is a balance between RANK‐RANKL and OPG activation. In addition, the RANK signaling pathway is crucial for differentiating and activating osteoclasts. Both OPG and RANKL are expressed by osteoblastic cells. The binding of RANKL to RANK promotes osteoclast activation, whereas the binding of OPG to RANKL inhibits this activation, promoting bone formation. This mechanism is triggered by the application of forces.^3^

In a rat study, Nishimura et al.^7^ described a relationship between movement acceleration and the application of vibrational forces, finding that utilization of a vibration device increased RANKL expression and osteoclastic activation. Likewise, Nakao et al.^8^ reported that RANKL expression of human periodontal ligament cells was stimulated by the application of intermittent vibratory forces. In contrast, Woodhouse et al.^9^ found that intermittent vibratory forces had no effect on the speed of initial orthodontic movement, measured as shifts in tooth position on study models; however, they did not investigate associated biochemical markers.

The aim of this study was to explore whether the application of intermittent vibratory force modifies RANKL and OPG concentrations in patients undergoing orthodontic treatment with clear aligners. The specific objective was to compare gingival crevicular fluid concentrations of RANKL and OPG among groups according to the application or not of Acceledent® treatment at different time points and the frequency of aligner changes.

## 2 MATERIAL AND METHODS

### 2.1 Trial design and changes after trial commencement

This three‐arm parallel randomized clinical trial (allocation ratio of 1:1:1) was approved by the hospital ethical committee for clinical research (ref: C.P.‐C.I: 15/313. ClinicalTrials.gov Identifier: NCT05316636). No changes were made to the original study protocol or methodology.

### 2.2 Participants, eligibility criteria, and setting

Participants were recruited from among patients attending the Clinic of a university department of orthodontics and two private orthodontic clinics. All patients were treated by the same orthodontist. Inclusion criteria were age between 21 and 50 years (inclusive) and treatment of malocclusion with Invisalign® (Align Technology, San Jose, CA), using at least 14 sets of aligners. Exclusion criteria were smoking habit, poor oral hygiene, the presence of periodontal disease (plaque indices >3 and community periodontal index of treatment needs >2; periodontal pockets >4 mm), or any other chronic or systemic diseases that could affect bone metabolism or inflammation, and previous or current receipt of medication that could influence bone metabolism (e.g., bisphosphonates).

### 2.3 Interventions

Patients who met the eligibility criteria were invited to participate in the study. All participants signed their informed consent.

Treatments were all carried out using Invisalign® aligners. Intermittent vibratory force was applied with an Acceledent® device (Acceledent Aura®, OrthoAccel Technologies, Bellaire, TX) for 20 min/day at an intensity of 30 Hz (25 g). The orthodontist evaluated Clincheck® (Align Technology) treatment simulations for all patients at an adjustment rate of 0.25 mm per aligner.

**FIGURE 1:**
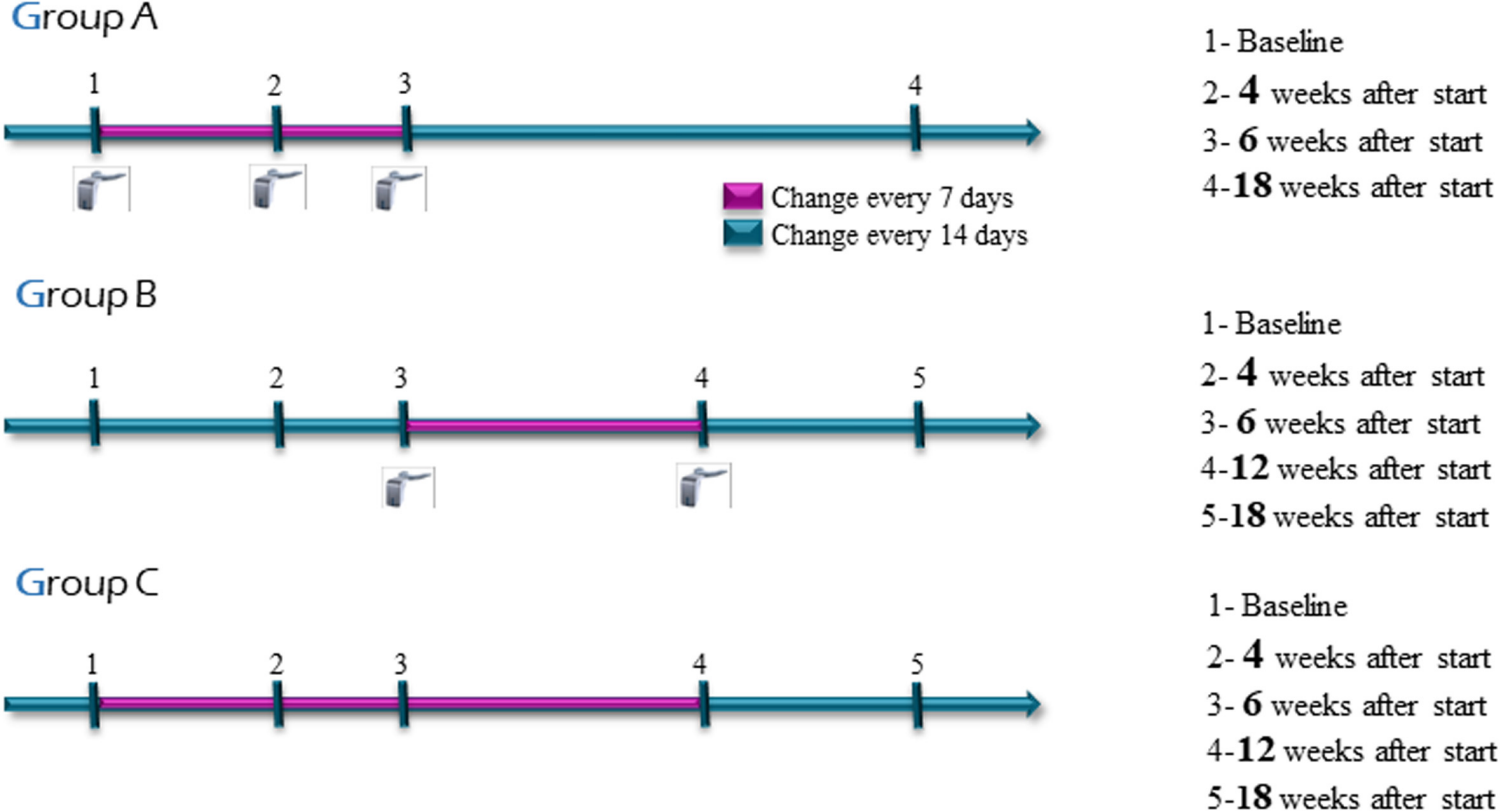
Interventions. Group distribution. Times of sampling/measurements

Patients were randomly assigned to one of three groups that underwent distinct interventions. Patients in Group A (*n* = 14) were instructed to use Acceledent® (20 min/day at 30 Hz, 0.3 N [25 g]) from treatment onset for 6 weeks, adjusting the aligners every 7 days for 6 weeks (as per Acceledent® protocol) and then every 14 days to the end of the 18‐week study period. Patients in Group B (*n* = 15) used Acceledent from treatment onset for 12 weeks, adjusting aligners every 14 days for the first 6 weeks and every 7 days for the next 6 weeks and every 14 days from weeks 12–18. Finally, control patients in Group C (*n* = 16) did not use the Acceledent® device and adjusted the aligners every 7 days for the first 12 weeks of treatment and then every 14 days from weeks 12–18. All patients had to wear the aligners for ≥22 h/day (Figure 1).

Comparisons were conducted between Group A (vibration with weekly change) and control Group C (weekly change without vibration from weeks 0 to 6), and between Group B (vibration with weekly change from week 6, i.e., after tooth movement onset) and Group C from 6 to 12 weeks. It was evaluated whether vibration had greater effect when applied at the earliest stage of tooth movement (Group A) or after the onset of movement (Group B). Results for Groups B and C from 0 to 6 weeks (no vibration forces in either group) were compared to examine the difference between changing aligners every 14 and 7 days in the absence of vibration. Between weeks 12 and 18, no group received vibration, and aligners were changed every 14 days.

### 2.4 Outcomes

The primary outcomes of this study were the gingival crevicular fluid concentrations of RANKL and OPG at different measurement time points. Secondary outcomes were Silness and Loe^10^ plaque index, Lobenne et al.^11^ modified gingival index, and bleeding on probing scores (in teeth under study and two adjacent teeth) at the same time points.

### 2.5 Crevicular fluid samples

Crevicular fluid samples were collected using absorbent paper tips 25 micron in diameter (Roeko®, Coltene, Cuyahoga Falls, OH).

A moving lower incisor was selected in each patient, and samples were always taken from the pressure point identified by Clincheck® with movement in vestibular direction. Samples were gathered at baseline (T0, pretreatment), 4 weeks (T1), 6 weeks (T2), 12 weeks (T3), and 18 weeks (T4) in all groups except for Group A (T1, T2, and T4). Photographic records and models were made at the same time points, and plaque and gingival index scores were measured after drawing the crevicular fluid sample.

Cotton rolls were used to isolate the tooth and clean the plaque remains, keeping the area dry and free of bleeding while the paper tip was inserted 1 mm into the gingival sulcus for 30 s. All samples were immediately frozen at −20°C and transferred to the UCM Research Laboratory for storage at −80°C with associated patient number and the time and date of sample collection.

### 2.6 Biochemical analysis

All 211 samples were thawed and extracted using a 200 μl solution containing phosphate buffer, phenylmethyl sulfonyl fluoride, and protease inhibitors. After incubation for 2 h at room temperature, samples were centrifuged for 10 min at 10,000 rpm, and ELISA kits were used to measure concentrations of soluble RANKL (Mybiosource®, San Diego, CA, USA, with sensitivity of 0.28 ng/L) and OPG (Elabscience® Biotechnology, Houston TX, USA, with sensitivity of 0.10 ng/ml) in duplicate, following the manufacturers’ instructions. The Prisma 8® program (Mybiosource®) was used to obtain the results, which were expressed as total concentration in μg/ml for both RANKL and OPG. All samples were destroyed after their analysis in accordance with Spanish legislation (Law 14/2007) on Biomedical Research.

### 2.7 Sample size calculation

The sample size estimation was based on the finding by Kawasaki et al. of a mean between‐group difference in tooth movement of 0.41 mm over 168 h with the application of force and their observation of a mean RANKL value during this period of 70,075 pg/μl (standard deviation [SD]: 27.6) versus 40,625 pg/μl (SD: 25.5) without force application. Consequently, an effect size (mean difference divided by its SD) of 1.108 was established, and a minimum sample of 14 patients was calculated for a statistical power of 80% and significance level of 5%.^6^

### 2.8 Randomization and blinding

A computer‐generated randomization list (GraphPad Software, Inc., USA) was used to randomly assign patients to study groups. Blinding was ensured by using preprepared sequentially numbered, opaque, sealed envelopes containing treatment allocation cards. A single researcher (CM) was responsible for opening the envelopes in sequence and implementing the randomization process. Neither patients nor orthodontist could be blinded to group membership because of the differences in treatment protocols. Nevertheless, assessments of clinical and laboratory outcomes were blinded because it was not possible to distinguish among the groups.

### 2.9 Statistical methods

The demographic characteristics of patients in each group were expressed as means with standard deviations and as frequencies. ANOVA and chi‐square tests were used to compare age and sex between groups. Mean values and standard deviations were calculated for RANKL and OPG concentrations and for clinical plaque and gingivitis index scores after verifying their normal distribution. Data were compared between and within groups by conducting a repeated measures ANOVA, with time and group as factors, and performing the Bonferroni correction. SPSS version 22 (IBM SPSS Inc., Armonk, NY) was used for statistical analyses, and the level of significance was set at *p* ≤ 0.05.

## 3 RESULTS

### 3.1 Participants

**FIGURE 2:**
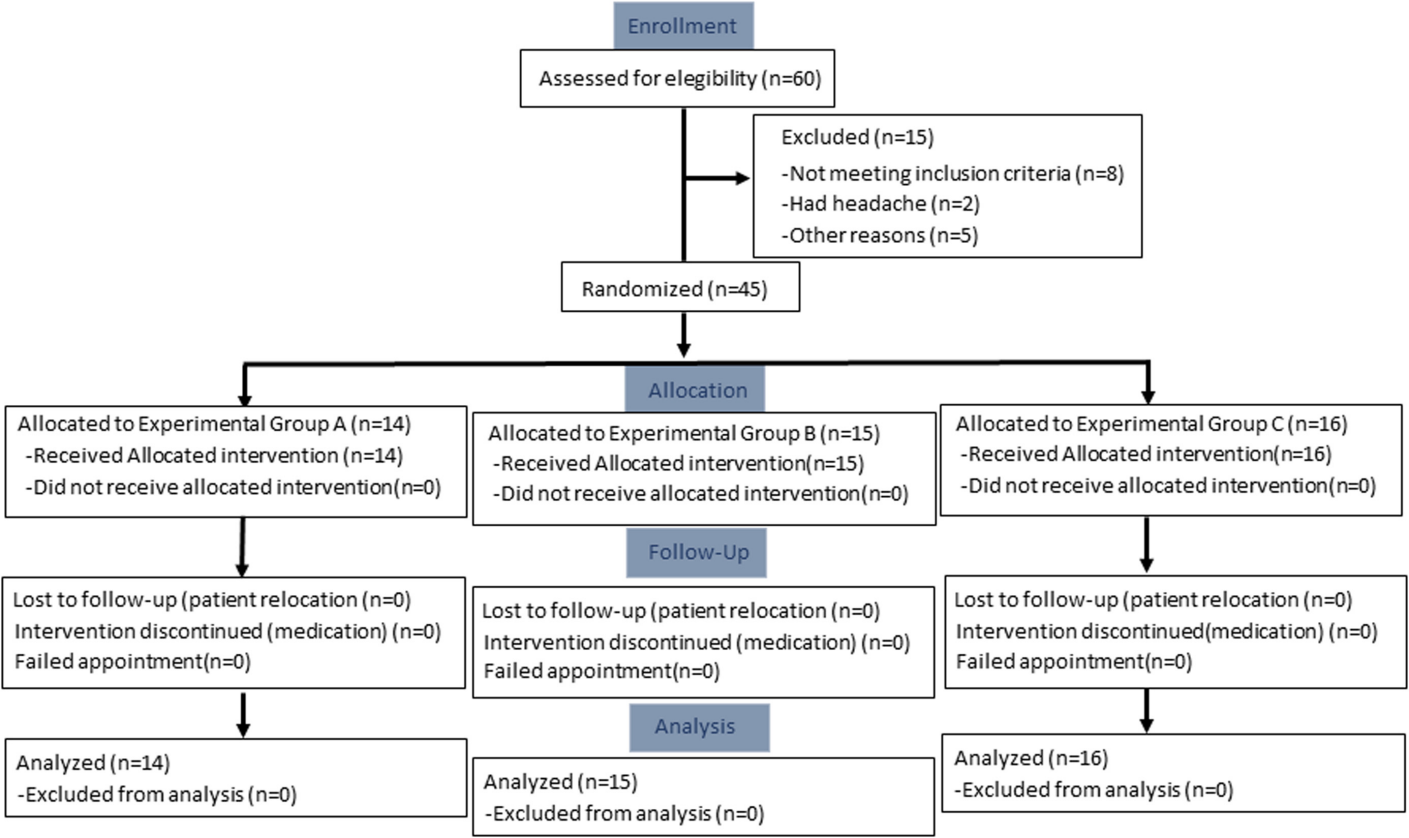
CONSORT flow chart of patients through the study

Out of 60 patients treated for malocclusion between September 2015 and October 2019, 15 were excluded from the study (see flow chart in Figure 2). Study eligibility criteria were met by 45 of the patients, who were randomly assigned to Groups A (*n* = 14), B (*n* = 15), or C (*n* = 16) (Figure 2). Table 1 lists the demographic and clinical characteristics of patients in the three groups. No between‐group differences were found in age (mean age = 30.82; SD = 8.33), sex (57.7% females, 42.2% males), gingival index score (range: 0.27–0.80), plaque index score (range: 0.1–0.55), or bleeding on probing score (range: 0.05–0.09).

**TABLE 1:** Distribution of study population by age and sex

Variable	Group A (*n*°=14)	Group B (*n*°=15)	Group C (*n*°=16)
Sex (*n*)			
Female	9	9	10
Male	5	6	6
Mean age (SD)	28.5 (9.55)	29.93 (6.9)	33.73 (8.54)
Mean GI (SD)	0.17 (0.35)	0.59 (0.8)	0.30 (0.27)
Mean PI (SD)	0.29 (0.41)	0.37 (0.55)	0.19 (0.22)
Mean BP (SD)	0.05 (0.05)	0.07 (0.09)	0.03 (0.05)

Abbreviations: BP, bleeding on probing; GI, gingival index; *n*, number of patients; PI, plaque index; SD, standard deviation.

### 3.2 Outcomes

#### 3.2.1 Primary outcomes: OPG and RANKL concentrations

##### Within‐group comparisons

###### OPG

**FIGURE 3:**
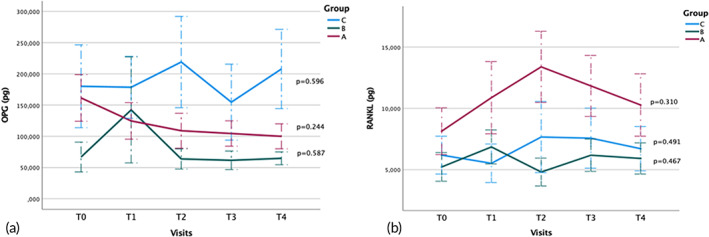
(a) Mean OPG measurements (in pg) at T0–T4 in Groups A, B, and C. (b) Mean RANKL measurements (in pg) at T0–T4 in Groups A, B, and C. *p* = statistical significance (repeated‐measures ANOVA). OPG, osteoprotegerin; RANKL, receptor activator of nuclear factor‐kappa B ligand

OPG concentrations did not significantly differ between time points in any study group (Figure 3). Although the differences were not statistically significant, mean OPG concentrations decreased over the treatment period in Group A (T0: 161.5 μg, SD = 144.45 μg; T1: 124.71 μg, SD = 113.40 μg; T2: 108.97 μg, SD = 108.41 μg; T4: 100.04 μg, SD =77.49 μg; *p* = 0.244); they increased with the application of vibration and then decreased in Group B (T0: 66.89 μg, SD = 92.34 μg; T1: 142.32 μg, SD = 328.70 μg; T2: 834.38 μg, SD = 2985.78 μg; T3: 2389.01 μg, SD = 9014.05 μg; T4: 64.77 μg, SD = 39.94, *p* = 0.587); and they increased after the first 4 weeks in Group C and then decreased after 6 weeks, observing a higher mean concentration when aligners were adjusted every 14 versus 7 days (T0: 180.20 μg, SD = 257.14 μg; T1: 178.58 μg, SD = 192.22 μg; T2: 219.03 μg, SD = 283.17 μg; T3: 154.73 μg, SD = 235.55 μg; T4: 207.85 μg, SD = 245.74 μg; *p* = 0.596).

###### RANKL

RANKL concentrations did not significantly differ among the three study groups at any time point (Figure 3). Although the differences were not statistically significant, mean RANKL concentrations rose during the first 6 weeks (vibration application and aligner adjustments every 7 days) in Group A and then decreased (T0: 8.14 μg, SD = 7.40 μg; T1: 10.88 μg, SD = 11.42 μg; T2: 13.39 μg, SD = 11.25 μg; T4: 10.28 μg, SD = 9.86 μg; *p* = 0.31). In Group B, they increased after changing the frequency of aligner adjustment to every 14 days and rose again with the application of vibration and aligner adjustments every 7 days (T0: 5.22 μg, SD = 4.52 μg; T1: 6.86 μg, SD = 5.38 μg; T2: 4.81 μg, SD = 4.41 μg; T3: 6.18 μg, SD = 0.09 μg; T4: 5.92 μg, SD = 4.93; *p* = 0.467). In Group C, they showed a small reduction when patients started adjusting aligners every 14 days (T0: 6.19 μg, SD = 5.98 μg; T1: 5.52 μg, SD = 6.10 μg; T2: 7.66 μg, SD = 11.25 μg; T3: 7.57 μg, SD = 9.50 μg; T4: 6.71 μg, SD = 0.02 μg; *p* = 0.491).

##### Between‐group comparisons

###### OPG

Table 2 exhibits the crevicular fluid OPG concentrations (in pg) obtained in Groups A, B, and C at different times (T1–T4), showing no statistically significant between‐group differences at any time point (T0: A = 161.50 μg, SD = 144.45 μg, B = 66.89 μg, SD = 92.34 μg, C = 180.20 μg, SD = 257.14 μg, *p* = 0.76; T1: A = 124.71 μg, SD = 113.40 μg, B = 142.32 μg, SD = 328.70 μg; C = 178.58 μg, SD = 192.22 μg, *p* = 0.447; T2: A = 108.97 μg, SD = 108.42 μg, B = 834.58 μg, SD = 2985.78 μg, C = 219.03 μg, SD = 283.17 μg, *p* = 0.832; T3: A = NA, SD = NA, B = 2389.01 μg, SD = 9014.05 μg, C = 154.73 μg, SD = 235.55 μg, *p* = 0.806; T4: A = 100.04 μg, SD = 77.49 μg, B = 64.77 μg, SD = 39.94 μg, C = 207.85 μg, SD = 45.74 μg, *p* = 0.699). Concentrations were nonsignificantly reduced when vibration was applied (mean = 11.97 μg SD = 216.55, p = 0.528) and appeared to be favored by aligner adjustment every 14 versus 7 days, although the difference was not statistically significant (mean = 251.33 μg, SD = 1727.81, *p* = 0.371).

**TABLE 2:** Intergroup comparison of OPG and RANKL values (pg), measured at different time points (T0–T4)

	OPG					RANKL				
	Mean	SD	Minimum	Maximum	*p*	Mean	SD	Minimum	Maximum	*p*
T0										
A	161.50	144.45	0.01	443.71		8.14	7.40	0.68	20.86	
B	66.89	92.34	0.22	367.92	0.76	5.22	4.52	0.39	11.76	0.496
C	180.20	257.14	6.91	942.70		6.19	5.98	0.43	17.54	
T1										
A	124.71	113.40	0.02	362.47		10.88	11.42	0.26	35.81	
B	142.32	328.70	2.68	1315.28	0.447	6.86	5.38	0.41	15.09	0.598
C	178.58	192.22	0.66	550.43		5.52	6.10	0.49	19.37	
T2										
A	108.97	108.42	1.60	388.09		13.39	11.25	0.21	31.98	
B	834.58	2985.78	7.19	11,625.46	0.832	4.81	4.41	0.31	12.44	0.103
C	219.03	283.17	1.60	978.72		7.66	11.25	0.07	39.61	
T3										
A	N/A	N/A	N/A	N/A		N/A	N/A	N/A	N/A	
B	2389.01	9014.05	3.69	34,972.3	0.806	6.18	5.09	0.24	14.02	0.683
C	154.73	235.55	0.63	742.92		7.57	9.50	0.37	29.19	
T4										
A	100.04	77.49	3.05	259.57		10.28	9.86	0.16	33.98	
B	64.77	39.94	7.51	155.28	0.699	5.92	4.93	0.39	12.30	0.658
C	207.85	245.74	1.09	828.65		6.71	7.02	0.31	19.89	

Abbreviations: N/A, not applicable; OPG, osteoprotegerin; *p*, statistical significance; RANKL, receptor activator of nuclear factor‐kappa B ligand; SD, standard deviation.

###### RANKL

As shown in Table 2, there were no statistically significant differences in RANKL concentrations among the different measurement time points. Although the differences were not statistically significant, RANKL concentrations were higher with the application of vibration (Groups A and B) and, if vibration was not applied, when aligners were adjusted every 14 days for the first 4 weeks and then every 7 days (Group C).Mean RANKL concentrations were as follows: T0: A = 8.14 μg, SD = 7.40 μg, B = 5.22 μg, SD = 4.52 μg, C = 6.19 μg, SD = 5.98 μg, *p* = 0.496; T1: A = 10.88 μg, SD = 11.42 μg, B = 16.89 μg, SD = 5.38 μg, C = 5.52 μg, SD = 6.10 μg, *p* = 0.598; T2: A = 13.39 μg, SD = 11.25 μg, B = 4.81 μg, SD = 4.41 μg, C = 7.66 μg, SD = 11.25 μg, *p* = 0.113; T3: A = NA, SD = NA, B = 6.18 μg, SD = 5.09 μg, C = 7.57 μg, SD = 9.50 μg, *p* = 0.683; T4: A = 10.28 μg, SD = 9.86 μg, B = 5.92 μg, SD = 4.93 μg, C = 6.71 μg, SD = 7.02 μg, *p* = 0.658.

#### 3.2.2 Secondary outcomes: plaque and gingival indices

No statistically significant between‐group or within‐group differences were found in gingival index, plaque index, or bleeding on probing scores (data not shown).

## 4 DISCUSSION

Various techniques have been developed to accelerate tooth movement and thereby reduce the duration of orthodontic treatments.^12^ A key research question is whether the association of higher tooth movement with increased RANKL concentrations and decreased OPG concentration found in rats can also be observed in humans.^4^, ^5^, ^6^

In this study on the effects of vibration with a specific device and the influence of aligner adjustment frequency, no statistically significant differences in crevicular fluid RANKL or OPG concentrations were found between treated and control teeth or between adjustments every 7 and 14 days. The lack of statistical significance may be due to the small sample size (*n* = 45), although this was estimated as adequate based on previously published results. It should also be borne in mind that the application of Acceledent® in a larger sample would have lengthened the study and increased research costs.

Differences among patients in the type of malocclusion were not considered in the present study because the magnitude of tooth movements was fixed and known, being determined by the aligner changes. Furthermore, concentrations of these biomarkers do not rise with increased force above a biological saturation point.^12^ This saturation point was found to be increased in rats after the application of vibration due to a direct effect on the periodontal ligament. This may exert an anabolic effect on tissues that are healthy or undergoing repair, as during the retention phase of orthodontic treatment, whereas the application of force and subsequent inflammatory reaction would have a catabolic effect.^12^

Although no statistically significant differences were found in crevicular fluid RANKL or OPG concentrations, a nonsignificant increase in the former and decrease in the latter were observed at various time points and associated with the application or non‐application of Acceledent®, suggesting possible changes in bone homeostasis. A nonsignificant rise in RANKL was observed when vibration was applied during the first 6 weeks of orthodontic treatment. In 2018, Judex and Pongkitwitoon^13^ also described a greater response at molecular and cellular levels after 3 days of vibration with Acceledent®, including a rise in RANKL concentrations, although these differences again failed to reach statistical significance. No change in RANKL concentrations were noted in the present study when vibration was applied after the onset of tooth movement, in line with the results published by Flórez‐Moreno et al.^5^; without applying vibration, they reported an increase after week 2 of treatment and a reduction at week 5, followed by an increase at week 8.^5^ They described a reduction in RANKL concentrations when aligners were changed every 14 versus 7 days, in line with the present findings. This may be attributable to binding to RANK during this phase (weeks 4–6) and/or to direct bone resorption during the longer time interval between aligner adjustments. Conversely, a change during this phase to aligner adjustments every 7 versus 14 days may increase RANKL concentrations, as suggested by the results obtained in controls.

Regarding OPG concentrations, a nonsignificant progressive reduction was observed when vibration was used from treatment onset, and an increase was recorded when it was not. In contrast, Alikhani reported that vibration has an anabolic effect on nonmoving teeth,^12^, ^13^, ^14^ although Yadav^15^ showed that the effect of vibration at 30 Hz may be more anabolic than catabolic, given the increase in OPG during phases when it should be reduced. These effects may vary according to the timing of vibration application and the length of interval between aligner changes at the start of treatment.^15^

Data were gathered on the concentrations of RANKL and OPG (pg) rather than the volume in each sample, applying the same dilution to homogenize the results. According to Leethanakul et al. knowledge of the volume of the sample or the concentration of protein is equally valid to determine changes in the concentration of these biomarkers.^16^

Comparisons with previous studies on the effects of vibration are limited because most examined clinical but not molecular changes. The purpose of the present investigation was to associate possible effects of vibration with increases and decreases in biomarker concentrations as a basis for future clinical research. To date, clinical studies have only examined the initial stages of tooth movement and have not obtained statistically significant results. Longer‐term research is hampered by the expectation that compliance with the vibration protocol would decline over time, reducing the reliability of results.^17^, ^18^, ^19^, ^20^ Besides the limited sample size, the lack of any statistically significant clinical or biomarker changes may be attributable to the vibration frequency used in the present investigation and most previous studies, given that frequencies above 90 Hz^14^, ^21^ were found to produce greater cell proliferation and gene expression in fibroblasts, thereby accelerating tooth movement.^21^

### 4.1 Adverse effects

No adverse effects were observed in any patient.

### 4.2 Limitations and generalizability

Study limitations include the relatively small sample size which may be responsible for the lack of statistically significant differences. Interpretation of these results should also take into account that the value of ranges of RANKL and OPG concentrations for periodontal diagnostics has not yet been validated. It would also have been useful to gather samples more frequently during initial phases of treatment and leave longer intervals after the third month to determine the cytokine response not only at the beginning but also at the end of treatment and in the retention phase. Protocols should ideally be standardized in future research in terms of the application of vibration, length of treatment, and vibration frequency, which can determine the cells that are activated. Finally, the periodontal effect of vibration should be studied in vivo in periodontally healthy individuals, as well as in patients with periodontal disease.

## 5 CONCLUSIONS

- The application of intermittent vibratory force using Acceledent® does not appear to significantly alter crevicular fluid RANKL and OPG concentrations in patients undergoing orthodontic treatment with Invisalign®.
- Although statistically significant results were not obtained, the application of vibration was associated with an increase in RANKL and a decrease in OPG concentrations, observing a greater increase in RANKL and decrease in OPG when applied at the initiation of treatment, before the onset of tooth movement.

## AUTHOR CONTRIBUTIONS

**Alicia Pérez Idarraga:** Conceptualization (equal); funding acquisition (equal); investigation (equal); methodology (equal); project administration (equal); resources (equal); supervision (equal); validation (equal); visualization (equal); writing – original draft (equal); writing – review and editing (equal). **Fara Yeste Ojeda:** Methodology (equal); supervision (equal); writing – review and editing (equal). **Leire Virto Ruiz:** Investigation (equal); supervision (equal); writing – review and editing (equal). **Miguel Lacasa Litner:** Investigation (equal); writing – review and editing (supporting). **Alberto Cacho Casado:** Investigation (equal); supervision (supporting); writing – review and editing (equal). **Conchita Martín:** Conceptualization (equal); data curation (equal); formal analysis (equal); funding acquisition (supporting); investigation (supporting); methodology (equal); project administration (supporting); software (equal); supervision (equal); validation (equal); writing – review and editing (supporting).

## FUNDING INFORMATION

This study was funded by the Spanish Society of Orthodontics (BIJC‐2017‐0001).

## CONFLICT OF INTEREST

The authors declare that they have no competing interests.

### PEER REVIEW

The peer review history for this article is available at https://publons.com/publon/10.1002/btm2.10491.

## ETHICS STATEMENT

Ethics Committee, San Carlos Clinical Hospital in Madrid, reference number C.P.‐C.I: 15/313, date of approval 06‐07‐2015.

## Data Availability

Data available on request from the authors.
